# Knowledge of clinicians/pediatricians about neonatal resuscitation in a tertiary care hospital

**DOI:** 10.12669/pjms.35.3.987

**Published:** 2019

**Authors:** Ayesha Muneer, Attia Bari, Arslan Haider, Agha Shabbir Ali

**Affiliations:** 1 *Dr. Ayesha Muneer, F.C.P.S. Assistant Professor of Pediatric Medicine , The Lahore General Hospital, Lahore, Pakistan*; 2 *Dr. Attia Bari, MCPS, DCH, F.C.P.S, MHPE. Associate Professor, Pediatric Medicine, The Children’s Hospital Lahore*; 3 *Dr. Arslan Haider, (Pediatric FCPS Part II resident), The Lahore General Hospital, Lahore, Pakistan*; 4 *Prof. Agha Shabbir Ali, MCPS, F.C.P.S. Pediatric Medicine, The Lahore General Hospital, Lahore, Pakistan*

**Keywords:** Neonates, Resuscitation, Health Professional, Knowledge

## Abstract

**Objective:**

To analyze the knowledge of the doctors dealing with pediatric patients about neonatal resuscitation.

**Methods:**

This was a cross sectional study conducted at The Lahore General Hospital over one year. Total 137 doctors related to pediatrics with different job descriptions were enrolled and requested to fill a questionnaire proforma regarding their knowledge about basic equipment required and about neonatal resuscitation steps. Data was entered and analyzed using SPSS 20.

**Results:**

Out of 137 participants, majority (71%) had >2 years of experience in pediatrics and 52.5% had higher postgraduate qualification. Neonatal resuscitation workshop was attended by 57% doctors. In resuscitation of newborns at the time of delivery, resuscitating doctors were assisted by nurse in 50%, by junior doctor in 35%, paramedic staff 11% and it was done by single doctor in 4% cases. Oxygen (central or O_2_ cylinder) and warmer facilities were available in 90% and 82% of health facilities respectively. Majority (86%) of participants were of view that every neonate must be attended at birth. Not a single doctor followed all the standard steps of neonatal resuscitation although 90% had knowledge about resuscitation equipment and common resuscitation drugs.

**Conclusion:**

Pediatric health care professionals had knowledge about neonatal resuscitation but there are gaps in the practical application. There is a strong need of frequent neonatal resuscitation workshops for improving neonatal outcomes.

## INTRODUCTION

The birth process is the most hazardous period of life and there are maximum number of deaths in this period as compared to deaths in all other phases of life.^1^ Approximately 10% of newborns require some form of resuscitation intervention immediately after birth.^2^ The ‘Helping Babies breath’ mainly focus on teaching neonatal resuscitation (NR) techniques in resource-limited settings.^3^,^4^ Providing good quality antenatal care and high quality resuscitation at birth by skilled birth attendants is a vital opportunity for all newborns to have a good start of life.^5^ Globally there is alarmingly high rate of death in newborn babies particularly among the world’s poorest countries and average newborn mortality rate in low-income countries is 27 deaths per 1000 births.^6^ A significant proportion of neonatal deaths in low and middle income countries are due to birth asphyxia and it accounts almost 25% of early neonatal deaths.^7^-^9^

NR is an essential component of maternal and child health services and is an inexpensive intervention by which many newborn lives can be saved. NR is defined as the set of interventions at the time of birth to support the establishment of breathing and circulation. The quality of NR and initial stabilization of newborn during first few minutes of birth has a significant effect on neonatal morbidity and mortality especially in high risk newborns like premature and low birth weight babies.^10^ Improper NR steps/Suboptimal resuscitation is a cause of millions of neonatal deaths and almost 99% of these deaths occur in resource limiting countries. A large number of these affected newborns develop complications such as cerebral palsy and cognitive impairment.^11^

If the health care providers are properly trained, then the outcome of these babies can be markedly improved. When births occur in health care facilities, it is a priority to ensure that all birth attendants are competent in newborn resuscitation. It is an ironic fact that most health care providers including doctors and nurses are inadequately trained.^12^ In order to create change and decrease neonatal mortality the quality improvement initiative is vital through effective neonatal resuscitation.^13^ There is scarcity of research on this topic in our country. In this context, we planned this study to identify the knowledge gaps or appropriateness among pediatric health care providers in NR at a tertiary care hospital, Lahore, Pakistan.

## METHODS

This was a descriptive observational cross-sectional study conducted at Lahore General Hospital, Lahore in 2017 over a period of one year in the department of Pediatric Medicine. After taking ethical approval from Institutional Review Board, a self-administered, close-ended questionnaire about the neonatal resuscitation knowledge was distributed to pediatric health care providers including pediatricians /doctors trained and working in neonatal unit or general pediatric ward and they were asked to complete questionnaire which was in two parts as; demographic information and assessment of their knowledge. The questionnaire consisted of simple questions based on basic steps done routinely during NR. Data regarding their age, qualification including postgraduate diploma or degree, experience in pediatrics, NR workshop attended and their Knowledge regarding basic steps of NR; clinical parameters of NR (following steps of resuscitation like Preparation, Safety, Shout for help and Stimulate) management (Airway, Breathing, Chest Compression, Drugs, Re-assessment), equipment required for resuscitation (20 cc Syringe, bulb sucker, suction catheter, nasogastric tube, mask of different sizes, ambu bag, laryngoscope with straight blade, endotracheal tubes of different sizes), drugs required for resuscitation (epinephrine, volume expenders, sodium bicarbonate, Naloxone, Dopamine Hydrochloride), and follow up assessment and care provided were recorded. The survey was anonymous as the names of the participants were kept confidential.

Data was analyzed using SPSS (Statistical Package for Social Sciences) Version 20. Frequencies, percentages were given for qualitative variables. The results are presented using tables.

## RESULTS

Out of 137 doctors’ 98 (71.5%) had > 2 years of experience in pediatrics and (72,52.5%) had highest postgraduate qualification (FCPS) followed by diploma who delivered neonatal resuscitation. Neonatal resuscitation workshop was attended by 78 (57%) doctors. In 69 (50%) deliveries doctor provided the resuscitation with the assistance of nurse and 6(4.4%) cases the resuscitation was done alone. Majority 118 (86%) were of the view that every neonate must be attended at birth by pediatrician. Details of neonatal resuscitation facility are shown in Table-I.

**Table-I:** Characteristics of the study participants and health facility for resuscitation (n=137)

Variables		Frequency (%)
Qualification	FCPS	72 (52.5)
	MBBS	12 (8.8)
	MCPS	17 (12.4)
	DCH	36 (26.2)
Facilities available/ equipment & monitors	Warmer	112 (82)
	Oxygen	124 (91)
	Monitor	20 (14)
	Ventilator	5 (4)
How important is Neonatal resuscitation?	Every baby must be attended	118 (86)
	Only high risk babies	19 (14)
Who helps you in resuscitation	Doctor	48 (35)
	Paramedic	14 (10.2)
	Nurse	69 (50.4)
	Alone	6 (4.4)
Experience in pediatrics	1 year	26 (19)
	1 to 2 year	13 (9.5)
	2 to 4 year	98 (71.5)
Neonatal workshop attended		78 (57)

All doctors did not follow all steps of resuscitation but 117 (85%) knew about the stimulus, drying and wrapping 110 (80%). It was quite worrisome that only 64 (47%) had the knowledge of how to maintain airway. Knowledge about common resuscitation drugs was 80-90% and majority >90% knew about the necessary equipment required for resuscitation (Table-II)

**Table-II:** Steps of Neonatal Resuscitation Followed by Pediatric Health Care Providers.

Things to do		Number (%)
Steps of resuscitation	Preparation	80 (58)
	Safety	90 (66)
	Shout for help	61 (45)
	How to dry and wrap	110 (80)
	Gentle tactile stimulation	117 (85)
	How to maintain airway	64 (47)
	Breathing	95 (69)
	Chest Compression	98 (72)
	Re-assessment	91 (66)
How frequently you check equipment required for resuscitation?	Every time	96 (70)
	Occasionally	41 (30)
How frequently do you check drugs required for resuscitation?	Regularly	114 (83)
	By Chance	23 (17)
Availability of priority items or equipment type	20 cc Syringe	115 (84)
	Bulb sucker	125 (91)
	Suction catheter	102 (75)
	NG tube	128 (93)
	Mask of different sizes	103 (75)
	Ambu bag with pressure valve	127 (93)
	Laryngoscope with straight blade	125 (91)
	Endotracheal tubes of different sizes	126 (92)
How frequently do you check drugs required for resuscitation?	Every time	110 (80)
	Occasionally	27 (20)
Awareness about availability of Essential Drugs	Epinephrine	126 (92)
	Volume Expenders	114 (83)
	Sodium bicarbonate	120 (88)
	Naloxone Hydrochloride	106 (77)
	Dopamine Hydrochloride	113 (83)

## DISCUSSION

To ensure the safety and health of neonates’, competency in NR is critical in the delivery rooms, neonatology units and pediatric intensive care units. Each year, millions of babies do not breathe immediately at birth, and among them the majority require basic neonatal resuscitation.^13^ When births occur in health care facilities, it is a priority to ensure that all birth attendants are competent in resuscitation.^5^ The results of our present study reflect the NR practices followed in tertiary care hospital where proper obstetric facilities for deliveries and neonatal unit including neonatal intensive care are available.

Despite the fact that our all participants belonged to pediatrics and questions were designed to assess simple base line knowledge, our participants were not able to mention all the steps of resuscitation accurately. Although majority 71.5% had >2 years of experience in pediatrics and had an experience of NR, only 45% were aware of the importance of shout for help and 72% accurately knew about the proper chest compression site and ventilation compression ratio. A similar study from India showed that only 35.4% of the participants managed to score above the minimum competency level of >85%. In their study, participants had >9 years of work experience.^14^ Our results are much better than this study as 52.5% of our participants had postgraduate qualification of FCPS. Both our and this study showed deficiency in knowledge of basic NR and many health personnel have limited knowledge of NR. These findings reflect the status of medical institutions in Pakistan that there are no regular refresher courses, or it is suboptimal training on essentials of NR.

Nurses are an integral part of team in delivery room and they must be well trained to provide initial resuscitation to newborns. In a study from India also a similar performance was reported where only 34% scored above 85% in NR steps.^15^ Training in NR for nurses, pediatric residents, and residents of obstetrics and gynecology must be emphasized as supported by a study done in Ethiopia showing substandard NR knowledge and skills.^16^ In our study, 57% had training in NR workshop which is comparable with a study from India which also showed that 57% received training in newborn care. In their study 48% had knowledge of positive pressure ventilation, 13% could provide chest compression or drugs during resuscitation.^17^ The important finding in this study was that most of the health workers did not had knowledge about NR. Our study showed much better results than this study findings as >60 percent showed appropriate knowledge of initial steps of resuscitation except of shouting for help and how to maintain airway. This difference is probably due to the difference that our hospital is a tertiary care hospital and majority had some form of postgraduate qualification. So, these findings showed that the knowledge about the NR could be changed by attending the refresher courses or workshops on NR.^18^ This is also supported by a study done by Bansal et al. from India in which the participant who underwent NR training were following correct practices as compared to those without the training.^19^

The primary steps in NR are very crucial in neonatal outcome and these include only the practices of suction to take a breath, drying, stimulating, keeping them warm, airway examination and assessment of breathing. These essential steps generally occur within the first few seconds of baby’s birth making rapid assessment of correct practice to save a life. A study from Pakistan published recently was conducted in obstetric department has shown similar results like ours, 58% of participants in this study showed correct initial steps for NR in labor room.^8^

The newborn babies who required extensive resuscitation should have ongoing post resuscitation assessment for next 24-48 hours after birth. Even those babies who respond well to initial simple resuscitation support may sometimes need further intervention in next few hours.^20^ In our study, only 66% had the perception of importance of reassessment of the baby after initial NR. Sometimes the gains from successful NR can be lost due to poor follow-up and aftercare and not attending to potential complications which may occur in next 24-48 hours.

### Limitations

The present study had several limitations. Only sampling pediatric doctors might mean that these results are not applicable to other health care professionals like nurses and obstetric doctors who are always at the forefront when a baby is delivered. Secondly the study participants were from only one tertiary care center of Lahore limiting the generalization of our results to others. More studies are required from other institutes as well and further studies to explore the reasons for the lack of understanding of NR to examine the all health care professional participating in newborn delivery.

## CONCLUSION

Pediatric health care professionals had knowledge about neonatal resuscitation but there are gaps in the knowledge which must be addressed. There is need to reassess the knowledge and skills of doctors and nurses providing NR and follow up by periodic NR refresher workshops and evaluation. Teaching on NR should be stressed during undergraduate and postgraduate medical education to ensure better neonatal outcome.

### Author’s Contribution

**AM:** Conceived idea, data collection.

**AB:** Main author, manuscript writing.

**AH:** Data management.

**ASA:** Critical review, final approval.
